# Human–Wildlife Conflicts: Does Origin Matter?

**DOI:** 10.3390/ani12202872

**Published:** 2022-10-21

**Authors:** Marcelo Hernán Cassini

**Affiliations:** Laboratorio de Biología del Comportamiento, IBYME, CONICET, Obligado 2490, Buenos Aires 1429, Argentina; mhcassini@yahoo.com.ar

**Keywords:** invasive species, conservation, wildlife management

## Abstract

**Simple Summary:**

Conflicts between humans and wildlife can occur with different types of problematic animals: native pests, dangerous native carnivores, invasive native species and invasive alien species. For conservation biology, the latter are the most damaging and must be managed differently than the first three types. I compared the damage done by native and introduced species in the United States using databases published on the Internet by Wildlife Services (WS), which depend upon the Animal and Plant Health Inspection Service of the Department of Agriculture. They receive thousands of complaints per year from the public and institutions due to wildlife damages, which they try to resolve. I found that human–wildlife conflict events were much more frequent with native species than with introduced ones. This pattern can be explained by at least three factors: because this organization biases their effort toward native fauna due to historical reasons, because people perceive the problems caused by native animals more, or because the impact of native species is greater than that of nonnative species. In any case, it seems reasonable to disregard the origin of species and try to resolve the most serious human–wildlife conflicts regardless of whether they are caused by native or introduced species.

**Abstract:**

Conservation biologists have divided wildlife in two antagonist categories—native and introduced populations—because they defend the hypothesis that the latter acquires or expresses harmful qualities that a population that remains in its original environment does not possess. Invasion biology has emerged as a branch of conservation biology dedicated exclusively to conflicts between introduced wildlife and human interest, including the protection of biodiversity. For invasion biology, the damage caused by native species is different and must be managed differently. However, the consensus around this native–introduced dichotomy is not universal, and a debate has intensified in recent years. The objective of this work was to compare the impacts of native and introduced species of terrestrial vertebrates of the United States using the dataset provided by Wildlife Services (WS), which depend upon the Animal and Plant Health Inspection Service of the Department of Agriculture. Annually, they receive thousands of reports and complaints of human–wildlife conflicts. I analyzed the WS databases and found, against expectations, that native species produce significantly more damage than nonnative ones, especially regarding damage to agriculture, property and health and safety. In the category of impacts on biodiversity and natural ecosystems, the differences were minor. I discuss several potential explanations of these patterns in the results. I also discuss the ecological foundations of the native–introduced dichotomy hypothesis.

## 1. Introduction

Conservation biology divides populations of the same animal species into two broad categories: native and introduced. The first are those that live within the original distribution range, while the second are those that live outside that range because they have been relocated. Conservation biology considers that the former must be protected to maintain biodiversity and ecosystem functions, while the latter must be controlled and, where possible, eradicated. When they spread (and produce damage or not), introduced species are called ‘invasive’ [1]. Conservation biologists’ beliefs are summarized in the following statements provided in a recent document of the International Union for Conservation of Nature: “it is expected that all introduced taxa will have an impact at some level, because by definition an alien taxon in a new environment has a nonzero impact” ([2], p. 9), and “lack of evidence of impacts must not be interpreted as lack of impact” ([2], p. 17).

Native species can also produce negative impacts on the environment, the economy and human health. Harmful native species can be classified into three different categories. Firstly, plague, pest or overabundant species are normally the result of two types of human environmental changes: agriculture and overhunting. For example, in mammals, agriculture has induced the increase in small mammal populations of a small number of species [3,4] while the overhunting of large carnivores has generated the overabundance of cervids and other ungulates [5]. Secondly, human–wildlife conflicts arise when large vertebrates, mainly carnivores, impact livestock husbandry and farming or the property and even the life of the inhabitants of the human settlements close to natural environments [6,7]. The third type is the so-called invasive-native species, i.e., native species that expand their geographical distribution, mainly due to climate change, and impact these new environments [8,9,10,11,12].

Many conservation biologists consider that the impact produced by native populations and those produced by introduced populations are different and thus require different management approaches [13]. They defend the hypothesis of a native-introduced dichotomy (NID) so that a population that originates from introduced individuals acquires or expresses harmful qualities that a population that remains in its original environment does not possess [14,15,16,17]. The prediction of this hypothesis is that, in each region or country, nonnative invasive species will produce significantly more impact than native pests, native conflict species or invasive native species.

The native–introduced dichotomy has generated a debate that continues. Davis et al. [18] published a seminal paper with a compelling title: ‘Don’t judge species on their origins’. The authors urged conservationists and land managers to focus much more on the functions of species rather than on whether they are native or introduced. In the following years, the debate has intensified [8,15,19,20,21]. Beyond the philosophical or ideological debate, various authors have focused on the analysis of the evidence that supports the hypothesis that nonnative species cause more damage than native ones. They tested the prediction of the NID hypothesis by conducting comparisons between the impact of native and introduced species mainly on biodiversity. The resulting publications show mixed results, with some of them supporting the hypothesis [15,21,22] and others not supporting it [23,24,25].

At least four different methods using different sources of information have been used: searches in the Web of Science [22,23], reviews of the information provided by the IUCN Red List of Endangered species [15], field experiments [25] and mesocosms, which allow for the very precise analysis of harmful ecological effects [26]. The objectives of this work were to compare the impacts of native and introduced species of terrestrial vertebrates in the United States using a new type of information and to discuss the NID hypothesis. I used the database provided by Wildlife Services (WS) that depend upon the Animal and Plant Health Inspection Service (APHIS) of the United States Department of Agriculture (USDA). Annually, they receive thousands of reports and complaints of human–wildlife conflicts involving both native and introduced species. In the discussion, I will not only analyze the results obtained with the WS database, but I will also brief analyze the ecological foundations of the NID hypothesis.

## 2. Materials and Methods

The USDA APHIS Wildlife Services’ mission is “to provide Federal leadership and expertise to resolve wildlife conflicts to allow people and wildlife to coexist” [27]. Biologists apply an integrated wildlife damage management approach to provide technical assistance and direct management operations in response to requests for assistance. Since 1996, they have published annual reports on their activities, including the number of animals that were controlled and the damage produced (https://www.aphis.usda.gov/aphis/ourfocus/wildlifedamage/sa_reports/sa_pdrs accessed on 5 July 2022). Since 2014, the reports have differentiated between invasive and noninvasive species.

To promote the vision of coexistence of people and wildlife, WS employees strive to reduce damage caused by wildlife to the lowest possible levels while at the same time reducing negative impacts on wildlife. In practice, this service begins to be rendered when the WS offices receive a request of assistance due to a wildlife damage problem. Then, WS personnel assess solutions from available, practical, cost-efficient, and environmentally and socially sound options. Finally, they help based on a wildlife damage-control strategy. When dealing with the protection of natural resources, including rare species, native wildlife and ecosystems, WS partners with Federal and State agencies, municipalities, organizations and private landowners.

In its webpage, the USDA APHIS provides what they call Program Data Reports C, which contain the complaints and reports of threats to resources by wildlife and occurrences of damage received by wildlife services. Each instance of assistance is defined as an ‘event’ and corresponds to those reported cases of damage or threats that include an actual reported value associated with the damage (WS handles other damage complaints in which a value for loss is not calculated or reported, which are not reported in the webpage). WS classifies damages into four categories: agricultural, natural, property and human health safety. They provide a detailed description of each category on their webpage: https://www.aphis.usda.gov/aphis/ourfocus/wildlifedamage/SA_Protected_Resources accessed on 5 July 2022. Each annual report worksheet contains four columns: resource category, resource damage or threatened, wildlife species causing damage and number of events. Since 2014, they have also provided information on the origin (native or introduced) of species. Therefore, I used C Reports from 2014 to 2021. I had to correct some typos (e.g., the same species named in singular and plural or the use of two tildes instead of one) and origin errors. I included in the analysis only terrestrial, wild and domestic feral vertebrates. I excluded 11,604 data points that corresponded to arthropods (196), fish (194), captive mammals (324) and unidentified animals (10,887).

Data did not have a normal distribution, so I used nonparametric statistics. I applied a chi-square test for observed versus expected frequency comparisons and contingency tables and a Mann–Whitney test for the comparison between native and introduced species. The alpha value was <0.05 in all tests.

## 3. Results

In the period of eight years, a total of 935,078 events on terrestrial vertebrate’s damages produced by a total of at least 630 species (nonnatives: n = 67, Supplementary materials) were registered on the website of the USDA APHIS WS.

**Figure 1:**
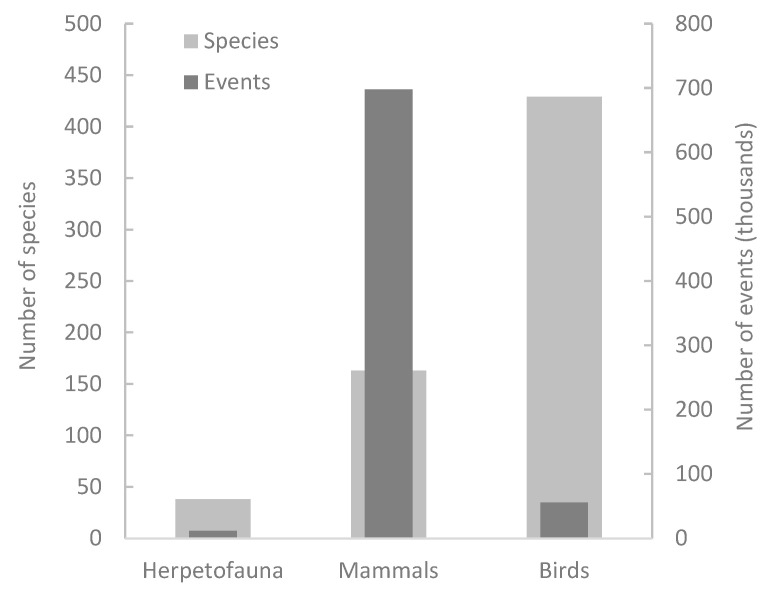
Mammals produced more conflict events. Birds had the greatest number of species reported, although events were significantly fewer (χ^2^ = 3366.1, *p* < 0.0001). ‘Herpetofauna’ refers to reptiles and amphibians. This graph is based on data of an 8-year period (2014–2021) provided by APHIS.

When comparing vertebrate taxa, mammals produced more significant conflicts than birds and reptiles/amphibians (χ^2^ = 3366.1, *p* < 0.0001) did, but birds involved a significantly greater number of species (χ^2^ = 379.8, *p* < 0.0001) (Figure 1).

**Figure 2:**
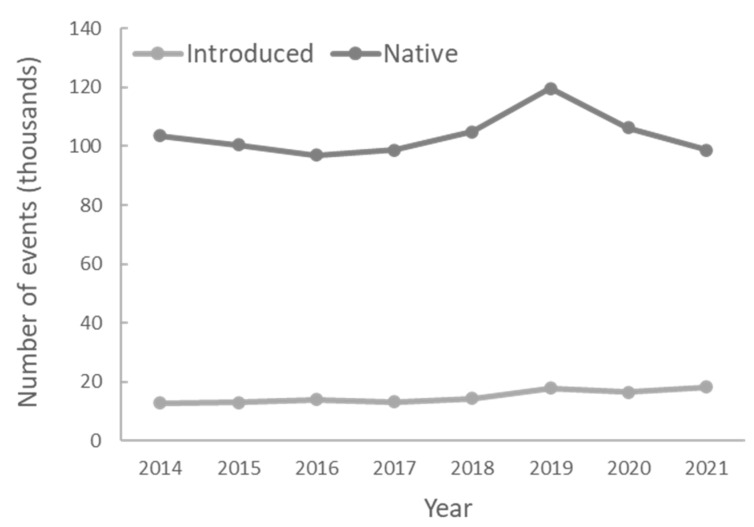
Number of events of conflicts with terrestrial vertebrates between 2014 and 2021.

**Figure 3:**
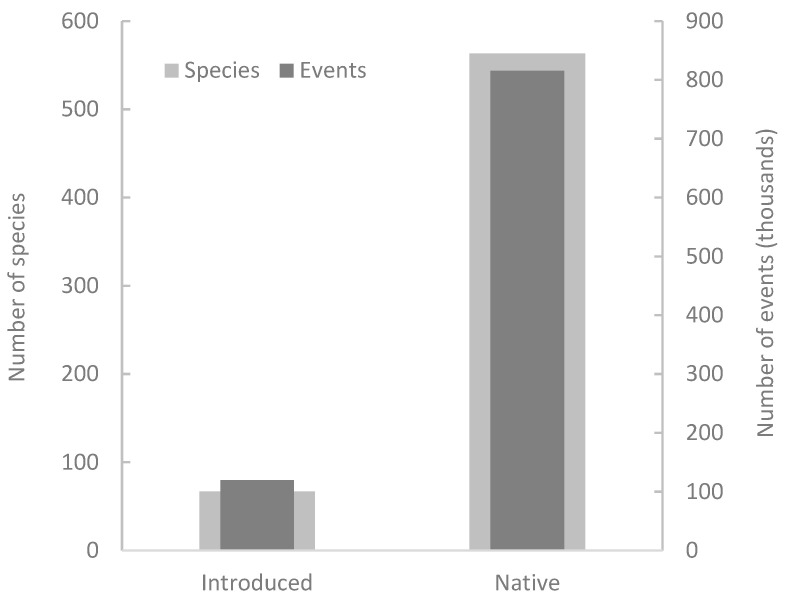
Number of events of conflict with terrestrial vertebrates and number of conflict species.

The number of events per year remained relatively constant throughout the period of eight years (Figure 2). Native species caused significantly more events of damage than did nonnative ones (χ^2^ = 548.2, *p* < 0.0001), and the number of species that produced them was also significantly higher (χ^2^ = 390.5, *p* < 0.0001) (Figure 3). However, there was no difference in the mean number of events per species between native (mean = 1448.3, SE = 395.9) and introduced (mean = 1786.2, SE = 1147.8) species (Mann–Whitney U test, U = 17,619.0, Z = −0.88, *p* = 0.38).

**Figure 4:**
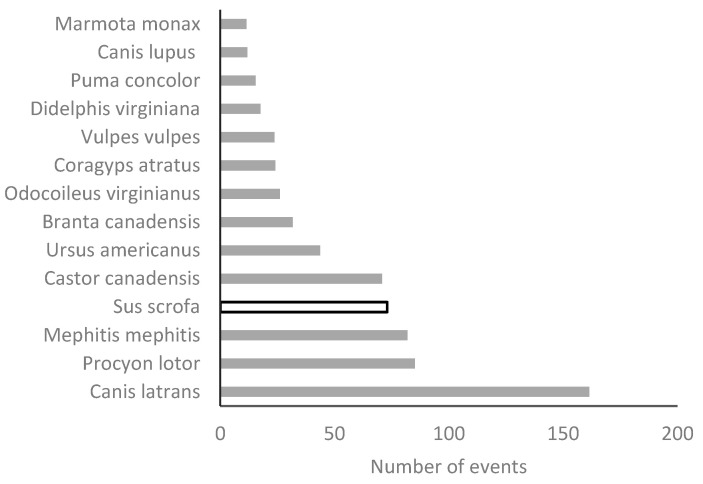
List of species that received the most complaints. The only introduced species of the list is represented with an open bar.

**Figure 5:**
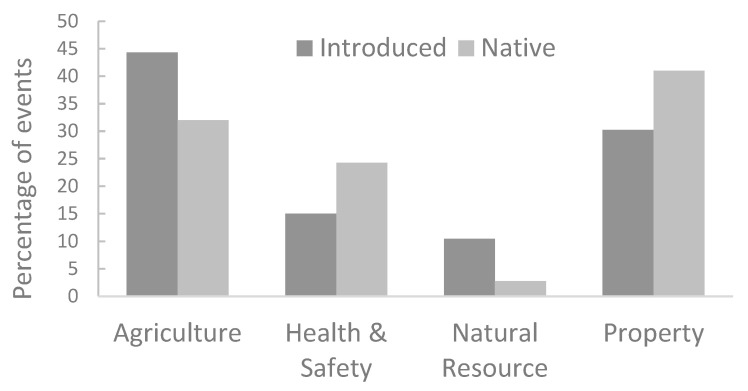
Percentage of events of impact to different types of resources, comparing native and introduced species.

Thirteen native and only one nonnative species appear among the species that received more than 10,000 complaints (Figure 4). In addition, 86% (12/14) of these species were mammals, and 64.3% (9/14) were carnivores. The species that received the highest number of complaints was the coyote *Canis latrans* (n = 161,491). Introduced wild boar *Sus scrofa* was the nonnative species on the list and appears in position four. When the different types of impacts were compared, the differences were highly significant both between impacts and between native and nonnative species (Figure 5). Native species produced more damage in the four categories, although the difference in natural resources was the lowest.

## 4. Discussion

The results of this review showed that the USDA APHIS Wildlife Services analyzed and solved significantly more cases of conflicts with native than with introduced wildlife. Although there were differences depending on the type of impact analyzed, the trend of the results was always the same. There are at least five possible explanations for this pattern, which I will briefly discuss in the following paragraphs.

A first possible explanation for this notable difference in favor of managing native species is that this wildlife service was traditionally dedicated to this task, while the commitment to controlling introduced species is more recent. Predator eradication with direct federal involvement began in the early 1900s and received direct public funding in 1915 [26,27,28]. For decades, lethal control of native wildlife was performed mainly to benefit livestock producers and to enhance game populations [29]. In this century, WS expanded areas of concern, including aviation safety, crop depredation, zoonotic disease and the safeguarding of rare, threatened and endangered wildlife species from the negative impacts of invasive ones [30]. At present, protecting natural resources (native species and ecosystems) is one of the five areas of concern of WS, who partner with Federal and State agencies, municipalities, organizations and private landowners (see the USDA webpage). Given the important status given in recent years to biological conservation and invasive species control, a bias toward more traditional areas of concern is to be expected.

Another explanation could be found in the way these databases are compiled. I do not belong to this organization, and my only link has been by its website. Therefore, I do not know the detailed procedures followed to transfer the information on the actual activities performed by WS employees to information loading in the data programs.

A third possible explanation is that the WS data published on C Report truly reflect what occurs in nature, i.e., that native species cause more damage than do introduced species. The use of complaints and reports is a method analogous to that applied in studies of morbidity impact on human populations. In order to estimate disease incidences, epidemiological studies frequently use indirect sources of data, such as insurance claims, accident and injury reports, clinic/hospital admissions, clinic/hospital discharge data and laboratory specimen analyses (review by [31]). Similarly, Klinkowski-Clark et al. [32] used animal control data to estimate the distribution of several species of mammals in Florida. They found that their results reflected those of other studies that used more conventional techniques.

The fourth hypothesis would be rooted in the very nature of the data being analyzed. As it was mentioned in the Introduction, studies based on bibliographic research and expert opinion showed mixed results, with some of them supporting the hypothesis [15,21,22] and others not finding differences between native and introduced species’ impacts [23,24,25]. However, none of these works found the pattern observed in the WS database, i.e., a clear bias toward a greater impact of native species. This could be because each type of study reflects different actors in the problem of wildlife damages: while the first type expresses the opinion of biologists that investigate impacts, the WS database reflects the opinion of the public and stakeholders who are directly affected by wildlife activities. Therefore, the analysis presented in this article could be criticized for being biased toward the interests of those who complain about wildlife impacts, rather than being objective research on those impacts.

The last explanation is probably the most parsimonious: there are many more native than nonnative species, and the same applies to total population numbers. For example, it is rare to introduce a large species, especially a large carnivore, and the US has a substantial number of large predators. This hypothesis appears to be supported by the result that there was no difference in the mean number of events per species between native and introduced species. This result suggests that, proportionally, complaints are equally dispersed and that the larger number of native species complaints is driven by their larger numbers and, therefore, greater likelihood of interaction. All these possible explanations are hypotheses that should be tested in future studies.

In the Introduction, I enunciated the NID hypothesis: What is the theoretical and empirical support to distinguish between impacts produced by introduced populations and impacts produced by native populations? Conservation biologists have proposed that native populations are safe from causing significant damage to natural resources by virtue of their roles and relationships within ecological and evolutionary systems and processes, as far as populations do not reach unnaturally high densities because of human activities [4,11]. In contrast, individuals outside their historical distribution area are no longer part of that natural diversity and lack those links with the rest of the ecosystem; thus, they are uncontrolled and represent a threat to invaded ecosystems [15,22]. Hui et al. [33] offered two concepts that organize the argumentative justifications of the hypothesis that introduced populations inevitably produce more damage than do native ones: invasibility and invasiveness. The first concept refers to a property of recipient ecosystems and involves the elucidation of features that determine their vulnerability to invasion, such as community diversity, composition and assembly. It proposes that introduced species find environments free of predators or competitors; thus, they can rapidly increase in distribution and abundance. For example, islands are considered more susceptible to biological invasions than the mainland because species have a greater chance of establishment when the recipient community lacks congeneric or ecologically similar species [34,35,36].

‘Invasiveness’ is the other concept introduced by Hui et al. [33] and refers to the propensity of introduced species to invade. These are traits that favor those groups of individuals that are translocated to new environments and can survive, establish themselves and then show high population growth and geographical expansion. These traits are typically related to a wide niche breadth, high reproductive capacity and high dispersal abilities [37,38,39,40]. Studies that seek to identify these characteristics use comparative metrics between successful and unsuccessful introduced species [33].

Abundant evidence indicates that habitat invasibility or extrinsic factors are more important determinants of introduction success in vertebrates than species invasiveness or intrinsic factors are [41,42,43,44,45]. While these concepts have been applied exclusively to ‘invasive’ species (hence their names), they can also be applied to native species in altered environments. In the Anthropocene, most ecosystems are degraded by human actions. This means that many native species inhabit environments in which some functional relationships are lost or new physical traits are found. Human impacts can increase the invasibility of native habitats, explaining why certain native species become a problem [6,7]. Native species can become pests due to habitat degradation that decreases predator or competition pressure (e.g., due to hunting pressure) or increases food availability (e.g., due to agriculture) without being transferred to exotic environments. Similarly, native species can become invasive-native species when climate change induces them to naturally colonize new environments [8,9,10,11,12].

If the cause that an introduced species becomes invasive and harmful is the ‘invasibility’ of new environments, then it would not be very different from the causes that determine that a native species becomes a pest. For example, rabbits introduced in an island without predators can dramatically increase their population size, impacting the vegetation of the island [46,47,48]. This is equivalent to the impact on native vegetation produced by a population of native rabbits when overhunting reduces or eliminates their predators [4]. In both cases, the invasibility of the environment determines species overabundances.

On the other hand, the concept of species invasiveness can also be applied to native species. As with the concepts of invasibility, invasiveness can also be applied to explain why some native species become superabundant and harm other native species or human activities while others do not. For example, native rodents are expected to become pests of agriculture because of their great reproductive rates [49]. Similarly, wild boar tend to turn into pests both in native and exotic habitats due to their wide diet niche [5].

Instead, there are two mechanisms that appear to be exclusive to introduced populations: one represents an advantage; the other, a drawback. The first is the eventual lack of adaptations by the inhabitants of the receiving environments due to the sudden appearance of a new species. Examples are native prey that would not have defenses against introduced predators and parasites that would not have infection mechanisms that are effective in the new animals (firstly described by [34]). In contrast to these eventual advantages, introduced animals must face new ecological relationships for which they do not necessarily have adaptations, thus finding themselves in worse competitive conditions than native populations. For example, European rabbits introduced to Argentinean Patagonia found a new predator, the minor grison *Galictis cuja*, which can enter rabbit burrows. A few years after their introduction, rabbits became the almost exclusive prey of grisons [50]. Despite this, I cannot exclude the fact that the introduced rabbits induced a disproportionate increase in the minor grison population, inducing a hyperpredation effect on native species in a manner similar to that observed by Cerri and colleagues after the introduction of Eastern cottontails in Italy [51]. To the best of my knowledge, there are no large-scale surveys that assess the degree of incidence of these two mechanisms as determinants of the success or failure of introductions.

## 5. Conclusions

Allocation of resources to conservation programs is normally limited, especially in peripheral countries, so environmental managers must generate decisions about which measures to prioritize and finance. In this context, the proposal by Davis et al. [18] of concentrating on the investigation of the most serious cases of animal impacts regardless of their origin seems more reasonable than generating a dubious dichotomy between native and nonnative species. This in no way means underestimating the importance of the damage that introduced species can cause, especially to biodiversity and natural ecosystems. Instead, it suggests avoiding the risk of investing in controlling species just because they are not native and not because of the degree of damage they do. Nor does it suggest for a moment to minimize the dramatic situation that many natural environments are going through. Quite the contrary, I believe that the seriousness of the situation warrants that the authorities become aware of the need to invest more resources into the conservation of biodiversity.

## Data Availability

Data can be found in the web page of the USDA APHIS Wildlife Services: https://www.aphis.usda.gov/aphis/ourfocus/wildlifedamage/sa_reports accessed on 24 July 2022.

## Supplementary Materials

The following supporting information can be downloaded at: https://www.mdpi.com/article/10.3390/ani12202872/s1, Table S1: List of species referred in APHIS tables.
