# Supplementary material for: Human–Wildlife Conflicts: Does Origin Matter?
Source: Animals (Basel). 2022 Oct 21;12(20):2872. doi: 10.3390/ani12202872 (PMC9598665; doi:10.3390/ani12202872)
Supplement: Supplementary file 1 [file animals-12-02872-s001.zip › animals-1940979-supplementary.pdf]

Table S1. List of species referred in APHIS tables.

Species	taxon	Native
Starlings, European	Birds	No
Pigeons, Feral (Rock)	Birds	No
Sparrows, House	Birds	No
Egrets, Cattle	Birds	No
Doves, Collared, Eurasian	Birds	No
Finches, House	Birds	No
Ducks, Feral	Birds	No
Swans, Mute	Birds	No
Sparrows, House/English	Birds	No
Mynas (All)	Birds	No
Chickens, Feral/Free Ranging	Birds	No
Pheasants, Ring-Necked	Birds	No
Doves, Zebra	Birds	No
Mannikins, Nutmeg	Birds	No
Doves, Spotted	Birds	No
Parakeets, Monk	Birds	No
Ducks, Feral Muscovy	Birds	No
Francolins, Black	Birds	No
Skylarks, Eurasian	Birds	No
Ducks, Muscovy	Birds	No
Cardinals, Red Crested	Birds	No
Fowl, Pea	Birds	No
Mannikins, Chestnut	Birds	No
Avadavats, Red	Birds	No
Bulbuls, Red-Vented	Birds	No
Waxbills, Common	Birds	No
Finches, Saffron	Birds	No
White-Eyes, Japanese	Birds	No
Sparrows, Eurasian Tree	Birds	No
Fowl, Guinea	Birds	No
Doves, Collared, Island (Philippine)	Birds	No
Fowl, Guinea, Feral Helmeted	Birds	No
Sparrows, Java	Birds	No
Bulbuls, Red-Whiskered	Birds	No
Silverbills, Warbling	Birds	No
Drongos, Black	Birds	No
Francolins, Erckel`s	Birds	No
Parakeets, Budgerigar	Birds	No
Parrots, Feral Amazon	Birds	No
Swans, Whooper	Birds	No
Waterfowl, Escaped/Hybrid/Exotic	Birds	No
Geese, Canada	Birds	Yes
Vultures, Black	Birds	Yes
Vultures, Turkey	Birds	Yes
Hawks, Red-Tailed	Birds	Yes
Gulls, Herring	Birds	Yes
Ravens, Common	Birds	Yes

Gulls, Ring-Billed	Birds	Yes
Crows, American	Birds	Yes
Doves, Mourning	Birds	Yes
Herons, Great Blue	Birds	Yes
Cormorants, Double-Crested	Birds	Yes
Ducks, Mallard	Birds	Yes
Blackbirds, Red-Winged	Birds	Yes
Cranes, Sandhill	Birds	Yes
Grackles, Common	Birds	Yes
Turkeys, Wild	Birds	Yes
Woodpeckers, Downy	Birds	Yes
Birds, unidentified	Birds	Yes
Killdeers	Birds	Yes
Woodpeckers, Hairy	Birds	Yes
Swallows, Barn	Birds	Yes
Egrets, Great	Birds	Yes
Hawks, Cooper`s	Birds	Yes
Ospreys	Birds	Yes
Meadowlarks, Eastern	Birds	Yes
Hawks, Harrier, Northern (Marsh Hawks)	Birds	Yes
Owls, Great Horned	Birds	Yes
Blackbirds, (Mixed Species)	Birds	Yes
Falcons, Kestrel, American	Birds	Yes
Robins, American	Birds	Yes
Eagles, Bald	Birds	Yes
Gulls, Black-Backed, Great	Birds	Yes
Cowbirds, Brown-Headed	Birds	Yes
Hawks, Red-Shouldered	Birds	Yes
Falcons, American Kestrels	Birds	Yes
Swallows, Cliff	Birds	Yes
Blackbirds, Yellow-Headed	Birds	Yes
Flickers, Northern	Birds	Yes
Gulls, Laughing	Birds	Yes
Coots, American	Birds	Yes
Meadowlarks, Western	Birds	Yes
Egrets, Snowy	Birds	Yes
Hawks, Sharp-Shinned	Birds	Yes
Larks, Horned	Birds	Yes
Owls, Snowy	Birds	Yes
Ducks, Teal, Blue-Winged	Birds	Yes
Pelicans, American White	Birds	Yes
Sandpipers, Upland	Birds	Yes
Pocket Gophers, (Other)	Birds	Yes
Falcons, Peregrine	Birds	Yes
Grackles, Great-Tailed	Birds	Yes
Ducks, Teal, Green-Winged	Birds	Yes
Owls, Common Barn	Birds	Yes

Ducks, American Black	Birds	Yes
Ducks, Whistling, Black-Bellied	Birds	Yes
Chipmunks, Eastern	Birds	Yes
Gulls, Franklin`s	Birds	Yes
Ducks, Shoveler, Northern	Birds	Yes
Mockingbirds, Northern	Birds	Yes
Ducks, Ring-Necked	Birds	Yes
Cardinals, Northern	Birds	Yes
Hawks, Swainson`s	Birds	Yes
Kingfishers, Belted	Birds	Yes
Herons, Night, Black-Crowned	Birds	Yes
Eagles, Golden	Birds	Yes
Pocket Gophers, Botta`s	Birds	Yes
Owls, Barred	Birds	Yes
Blackbirds, Brewer`s	Birds	Yes
Herons, Green	Birds	Yes
Ducks, Gadwall	Birds	Yes
Woodpeckers, Pileated	Birds	Yes
Ibises, White	Birds	Yes
Geese, Snow, Lesser	Birds	Yes
Jays, Blue	Birds	Yes
Owls, Short-Eared	Birds	Yes
Caracaras, Northern Crested	Birds	Yes
Hawks, Rough-Legged	Birds	Yes
Gulls, California	Birds	Yes
Ducks, Wigeon, American	Birds	Yes
Swallows, Tree	Birds	Yes
Doves, White-Winged	Birds	Yes
Fishers	Birds	Yes
Grebes, Pied-Billed	Birds	Yes
Buntings, Snow	Birds	Yes
Gulls, Bonaparte`s	Birds	Yes
Sparrows, Savannah	Birds	Yes
Ducks, Scaup, Lesser	Birds	Yes
Gulls, Western	Birds	Yes
Ducks, Wood	Birds	Yes
Ducks, Pintail, Northern	Birds	Yes
Herons, Little Blue	Birds	Yes
Ducks, Bufflehead	Birds	Yes
Kites, Mississippi	Birds	Yes
Ducks, Goldeneye, Common	Birds	Yes
Yellowlegs, Greater	Birds	Yes
Plovers, Golden, Pacific	Birds	Yes
Ducks, Canvasback	Birds	Yes
Woodpeckers, Red-Headed	Birds	Yes
Sandpipers, Least	Birds	Yes
Terns, Caspian	Birds	Yes
Ducks, Merganser Common	Birds	Yes

Terns, Least	Birds	Yes
Pelicans, Brown	Birds	Yes
Ducks, Redhead	Birds	Yes
Ducks, Merganser, Hooded	Birds	Yes
Herons, Night, Yellow-Crowned	Birds	Yes
Magpies, Black-Billed	Birds	Yes
Sandpipers, Semipalmated	Birds	Yes
Ducks, Mottled	Birds	Yes
Herons, Tricolored	Birds	Yes
Skimmers, Black	Birds	Yes
Woodpeckers, Red-Bellied	Birds	Yes
Cormorants, Neotropic (Olivaceous)	Birds	Yes
Ducks, Merganser, Red-Breasted	Birds	Yes
Ducks, Teal, Cinnamon	Birds	Yes
Plovers, Black-Bellied	Birds	Yes
Hawks, Broad-Winged	Birds	Yes
Yellowlegs, Lesser	Birds	Yes
Flycatchers, Scissor-Tailed	Birds	Yes
Ducks, Whistling, Fulvous	Birds	Yes
Swifts (unadentify)	Birds	Yes
Geese, Brant, Atlantic	Birds	Yes
Plovers, Semipalmated	Birds	Yes
Terns, Forster`s	Birds	Yes
Kingbirds, Western	Birds	Yes
Sanderlings	Birds	Yes
Kingbirds, Eastern	Birds	Yes
Waxwings, Cedar	Birds	Yes
Roadrunners	Birds	Yes
Whimbrels	Birds	Yes
Anhingas	Birds	Yes
Curlews, Long-Billed	Birds	Yes
Storks, Wood	Birds	Yes
Geese, White-Fronted, Greater	Birds	Yes
Geese, Snow, Greater	Birds	Yes
Sandpipers, Buff-Breasted	Birds	Yes
Ringtails	Birds	Yes
Snipes, Wilson`s	Birds	Yes
Dowitchers, Long-Billed	Birds	Yes
Ducks, Ruddy	Birds	Yes
Swallows, Bank	Birds	Yes
Woodpeckers, Golden-Fronted	Birds	Yes
Crows, Fish	Birds	Yes
Quail, Northern Bobwhite	Birds	Yes
Pocket Gophers, Northern	Birds	Yes
Bitterns, Yellow	Birds	Yes
Owls, Burrowing	Birds	Yes
Owls, Eastern Screech	Birds	Yes
Swifts, Chimney	Birds	Yes

Caracaras	Birds	Yes
Martins, Purple	Birds	Yes
Pocket Gophers, Plains	Birds	Yes
Pocket Gophers, Yellow-Faced	Birds	Yes
Catbirds, Gray	Birds	Yes
Willets	Birds	Yes
Falcons, Merlin	Birds	Yes
Juncos, Dark-Eyed	Birds	Yes
Nighthawks (All)	Birds	Yes
Ducks, Northern Pintail	Birds	Yes
Stilts, Black-Necked	Birds	Yes
Albatrosses, Laysan	Birds	Yes
Terns, Common	Birds	Yes
Turnstones, Ruddy	Birds	Yes
Sandpipers, Pectoral	Birds	Yes
Sandpipers, Solitary	Birds	Yes
Woodpeckers, Sapsuckers, Yellow-Bellied	Birds	Yes
Shrikes, Loggerhead	Birds	Yes
Nighthawks, Common	Birds	Yes
Grackles, Boat-Tailed	Birds	Yes
Pipits, American	Birds	Yes
Bitterns, American	Birds	Yes
Bobolinks	Birds	Yes
Frigatebirds, Greater	Birds	Yes
Ducks, Hawaiian (T/E)	Birds	Yes
Wrens, House	Birds	Yes
Loons, Common	Birds	Yes
Nuthatches, White-Breasted	Birds	Yes
Falcons, Prairie	Birds	Yes
Owls, Long-Eared	Birds	Yes
Blackbirds, Rusty	Birds	Yes
Finches, Purple	Birds	Yes
Geese, Cackling	Birds	Yes
Sparrows, Field	Birds	Yes
Woodcock, American	Birds	Yes
Gulls, Glaucous-Winged	Birds	Yes
Ibises, Glossy	Birds	Yes
Ducks, Scaup, Greater	Birds	Yes
Warblers, Yellow-Rumped	Birds	Yes
Hawks, Goshawk, Northern	Birds	Yes
Chickadee, Black-Capped	Birds	Yes
Quail (Other)	Birds	Yes
Ducks, Northern Shoveler	Birds	Yes
Sparrows, White-Throated	Birds	Yes
Quail, Gambel`s	Birds	Yes
Dowitchers, Short-Billed	Birds	Yes
Ducks, Eider, Common	Birds	Yes

Hawks, Ferruginous	Birds	Yes
Sparrows, Lincoln`s	Birds	Yes
Phoebes, Eastern	Birds	Yes
Longspurs, Lapland	Birds	Yes
Plovers, Golden, American	Birds	Yes
Swans, Tundra	Birds	Yes
Sparrows, Fox	Birds	Yes
Swallows, Northern Rough-Winged	Birds	Yes
Thrushes, Hermit	Birds	Yes
Dickcissels	Birds	Yes
Swans, Trumpeter	Birds	Yes
Dunlins	Birds	Yes
Hawks, Harris	Birds	Yes
Sparrows, Grasshopper	Birds	Yes
Thrashers, Brown	Birds	Yes
Thrushes, Wood	Birds	Yes
Gulls, Heermann`s	Birds	Yes
Sparrows, Chipping	Birds	Yes
Geese, Aleutian Cackling	Birds	Yes
Pocket Gophers, Desert	Birds	Yes
Ducks, Goldeneye, Barrow`s	Birds	Yes
Grouse, Ruffed	Birds	Yes
Ibises, White-Faced	Birds	Yes
Grebes, Western	Birds	Yes
Hummingbirds, Ruby-Throated	Birds	Yes
Oystercatchers, American	Birds	Yes
Wrens, Marsh	Birds	Yes
Grebes, Horned	Birds	Yes
Sparrows, American Tree	Birds	Yes
Grosbeaks, Rose-Breasted	Birds	Yes
Gulls, Black-Backed, Lesser	Birds	Yes
Gulls, Glaucous	Birds	Yes
Ovenbirds	Birds	Yes
Phoebes, Say`s	Birds	Yes
Pocket Gophers, Western	Birds	Yes
Sparrows, Vesper	Birds	Yes
Bluebirds, Mountain	Birds	Yes
Sandpipers, Spotted	Birds	Yes
Terns, Royal	Birds	Yes
Longspurs, Smith`s	Birds	Yes
Warblers, Wilsons	Birds	Yes
Terns, Gull-Billed	Birds	Yes
Wrens, Sedge	Birds	Yes
Sparrows, Lark	Birds	Yes
Warblers, Common, Yellowthroat	Birds	Yes
Flycatchers, Pacific-Slope	Birds	Yes
Geese, Hawaiian (T/E)	Birds	Yes
Orioles, Northern	Birds	Yes

Pocket Gophers, Texas	Birds	Yes
Sandpipers, Western	Birds	Yes
Terns, Black	Birds	Yes
Blackbirds, Tri-Colored	Birds	Yes
Buntings, Lark	Birds	Yes
Geese, Ross`s	Birds	Yes
Thrushes, Swainson`s	Birds	Yes
Vireos, Warbling	Birds	Yes
Gnatcatchers, Blue-Gray	Birds	Yes
Gulls, Mew	Birds	Yes
Stilts, Hawaiian (T/E)	Birds	Yes
Swallows, Violet-Green	Birds	Yes
Terns, Roseate (T/E)	Birds	Yes
Titmouse, Tufted	Birds	Yes
Warblers, Yellow	Birds	Yes
Swallows, Cave	Birds	Yes
Warblers, Orange-Crowned	Birds	Yes
Bluebirds, Western	Birds	Yes
Gulls, Iceland	Birds	Yes
Orioles, Baltimore	Birds	Yes
Partridge, Gray	Birds	Yes
Sandpipers, Common	Birds	Yes
Shrikes (Other)	Birds	Yes
Sparrows, Golden-Crowned	Birds	Yes
Swifts, White-Throated	Birds	Yes
Thrashers, Curve-Billed	Birds	Yes
Vireos, Red-Eyed	Birds	Yes
Boobys, Brown	Birds	Yes
Cranes, Whooping	Birds	Yes
Dippers, American	Birds	Yes
Geese, Brant, Black	Birds	Yes
Grebes, Eared	Birds	Yes
Hawks, Zone Tailed	Birds	Yes
Jays, Steller`s	Birds	Yes
Owls, Northern Saw-Whet	Birds	Yes
Pigeons, Band-Tailed	Birds	Yes
Sandpipers, Baird`s	Birds	Yes
Tanager, Scarlet	Birds	Yes
Tanagers, Western	Birds	Yes
Woodpeckers, Ladder-Backed	Birds	Yes
Grosbeaks, Black-Headed	Birds	Yes
Kites, Swallow-Tailed	Birds	Yes
Loons, Red-Throated	Birds	Yes
Pocket Gophers, Baird`s	Birds	Yes
Sparrows, Black-Chinned	Birds	Yes
Spoonbills, Roseate	Birds	Yes
Warblers, Hermit	Birds	Yes
Hawks, White-Tailed	Birds	Yes

Loons, Pacific	Birds	Yes
Sandpipers, Stilt	Birds	Yes
Shearwaters (Other)	Birds	Yes
Sparrows, Brewer`s	Birds	Yes
Geese, White-Fronted, Lesser	Birds	Yes
Owls, Western Screech	Birds	Yes
Sandpipers, Wood	Birds	Yes
Shearwaters, Wedge-Tailed	Birds	Yes
Terns, Common Fairy	Birds	Yes
Woodpeckers, Red-Cockaded (T/E)	Birds	Yes
Coots, Hawaiian (T/E)	Birds	Yes
Curlews, Bristle-Thighed	Birds	Yes
Ducks, Scoter, White-Winged	Birds	Yes
Flycatcher, Ash-Throated	Birds	Yes
Grouse, Sharp-Tailed	Birds	Yes
Hawks, Hawaiian (T/E)	Birds	Yes
Herons, Reef, Pacific	Birds	Yes
Hummingbirds, Anna`s	Birds	Yes
Jays, Scrub	Birds	Yes
Kinglet, Ruby-Crowned	Birds	Yes
Kites, White-Tailed	Birds	Yes
Nighthawks, Lesser	Birds	Yes
Nuthatches, Red-Breasted	Birds	Yes
Orioles, Bullock`s	Birds	Yes
Petrels, Hawaiian Dark-Rumped (T/E)	Birds	Yes
Poorwill, Common	Birds	Yes
Redpolls, Common	Birds	Yes
Sparrows, Black-Throated	Birds	Yes
Tattlers, Wandering	Birds	Yes
Thrushes, Varied	Birds	Yes
Woodpeckers, Sapsuckers, Red-Naped	Birds	Yes
Buntings, Indigo	Birds	Yes
Grebes, Eared (Black-Necked)	Birds	Yes
Jays, Scrub, California	Birds	Yes
Nuthatches, Pygmy	Birds	Yes
Orioles, Hooded	Birds	Yes
Owls, Great Gray	Birds	Yes
Pocket Gophers, Knox Jone`s	Birds	Yes
Towhees, Canyon	Birds	Yes
Warblers,Townsend`s	Birds	Yes
Wrens, Cactus	Birds	Yes
Chukars	Birds	Yes
Cormorants, Pelagic	Birds	Yes
Crawfish, Red Swamp	Birds	Yes
Ducks, Scoter, Surf	Birds	Yes
Geese, Emperor	Birds	Yes
Godwits, Marbled	Birds	Yes
Gulls, Thayer`s	Birds	Yes

Hummingbirds, Black-Chinned	Birds	Yes
Hummingbirds, Rufous	Birds	Yes
Jaegers, Long-Tailed	Birds	Yes
Moorhens, Common	Birds	Yes
Nightjars, Whippoorwill, Eastern	Birds	Yes
Phalaropes, Red	Birds	Yes
Phalaropes, Red-Necked	Birds	Yes
Phoebes, Black	Birds	Yes
Pikeminnows, Northern	Birds	Yes
Plovers, Mountain	Birds	Yes
Plovers, Snowy	Birds	Yes
Plovers, Western Snowy	Birds	Yes
Quail, California	Birds	Yes
Rails, Virginia	Birds	Yes
Siskins, Pine	Birds	Yes
Sora	Birds	Yes
Terns, Whiskered	Birds	Yes
Towhees, Spotted	Birds	Yes
Turnstones, Black	Birds	Yes
Warblers, Blackpoll	Birds	Yes
Warblers, Black-Throated Gray	Birds	Yes
Warblers, Nashville	Birds	Yes
Buntings, Lazuli	Birds	Yes
Chipmunks, Least	Birds	Yes
Cormorants, Brandts	Birds	Yes
Crossbills, White-Winged	Birds	Yes
Doves, Ground, Common	Birds	Yes
Doves, Inca	Birds	Yes
Ducks, Scoter, Black	Birds	Yes
Finches, Gray Crowned Rosy	Birds	Yes
Goldfinches, Lawrence`s	Birds	Yes
Goldfinches, Lesser	Birds	Yes
Grebes, Clarks	Birds	Yes
Grosbeaks, Evening	Birds	Yes
Grouse, Sage, Greater	Birds	Yes
Hummingbirds, Costa``s	Birds	Yes
Jays, Gray	Birds	Yes
Kinglets, Golden-Crowned	Birds	Yes
Owls, Northern Pygmy	Birds	Yes
Pocket Gophers, Attwater`s	Birds	Yes
Pocket Gophers, Camas	Birds	Yes
Pocket Gophers, Townsend`s	Birds	Yes
Snipes, Common	Birds	Yes
Sparrows, Sage	Birds	Yes
Towhees, Green-Tailed	Birds	Yes
Verdins	Birds	Yes
Woodpeckers, Lewis`s	Birds	Yes
Wrens, Canyon	Birds	Yes

Swine, Feral	Mammals	No
Dogs, Feral, Free-Ranging And Hybrids	Mammals	No
Cats, Feral/Free Ranging	Mammals	No
Nutrias	Mammals	No
Rats, Norway	Mammals	No
Rats, Black (Roof)	Mammals	No
Bats, Common Vampire	Mammals	No
Mice, House	Mammals	No
Rabbits, Feral	Mammals	No
Cattle, Feral	Mammals	No
Antelope, Nilgai	Mammals	No
Deer, Fallow	Mammals	No
Horses, Feral	Mammals	No
Sheep, Feral, Free-Ranging And Exotics	Mammals	No
Deer, Sambar (Philippine)	Mammals	No
Mongooses, Indian	Mammals	No
Burros, Feral	Mammals	No
Ferrets, European	Mammals	No
Deer, Axis	Mammals	No
Rats, Polynesian	Mammals	No
Goats, Mountain	Mammals	No
Coyotes	Mammals	Yes
Raccoons	Mammals	Yes
Skunks, Striped	Mammals	Yes
Beavers	Mammals	Yes
Bears, Black	Mammals	Yes
Deer, White-Tailed (Wild)	Mammals	Yes
Foxes, Red	Mammals	Yes
Opossums, Virginia	Mammals	Yes
Lions, Mountain (Cougar)	Mammals	Yes
Wolves, Gray/Timber	Mammals	Yes
Woodchucks	Mammals	Yes
Bobcats	Mammals	Yes
Foxes, Gray	Mammals	Yes
Squirrels, Eastern Gray	Mammals	Yes
Bears, Grizzly	Mammals	Yes
Prairie Dogs, Gunnison`s	Mammals	Yes
Marmots/Woodchucks (All)	Mammals	Yes
Prairie Dogs, Black-Tailed	Mammals	Yes
Bats (unidentified)	Mammals	Yes
Muskrats	Mammals	Yes
Bears, Louisiana Black	Mammals	Yes
Badgers	Mammals	Yes
Bats, Brown, Big	Mammals	Yes
Wolves, Mexican Gray	Mammals	Yes
Squirrels, Ground, California	Mammals	Yes
Peccaries, Collared (Javelina)	Mammals	Yes
Porcupines	Mammals	Yes

Otters, River	Mammals	Yes
Squirrels, Rock	Mammals	Yes
Armadillos, Nine-Banded	Mammals	Yes
Squirrels, Fox	Mammals	Yes
Mammal, unidentified	Mammals	Yes
Rabbits, Cottontails, Desert	Mammals	Yes
Rabbits, Cottontails, Eastern	Mammals	Yes
Rabbits, Cottontail	Mammals	Yes
Bats, Brown, Little	Mammals	Yes
Hares, Jackrabbits, Black-Tailed	Mammals	Yes
Squirrels, Ground (Other)	Mammals	Yes
Deer, Mule	Mammals	Yes
Skunks, Hooded	Mammals	Yes
Minks	Mammals	Yes
Moles (unadentified)	Mammals	Yes
Elk, Wapiti (Wild)	Mammals	Yes
Squirrels, Western Gray	Mammals	Yes
Bats, Brazilian (Mexican) Free-Tailed	Mammals	Yes
Marmots, Yellow-Bellied	Mammals	Yes
Mice, Deer (unadentified)	Mammals	Yes
Deer, Black-Tailed	Mammals	Yes
Skunks, Spotted	Mammals	Yes
Voles (Other)	Mammals	Yes
Squirrels, Flying (All)	Mammals	Yes
Skunks, Hog-Nosed	Mammals	Yes
Squirrels, Abert`s	Mammals	Yes
Squirrels, Ground, Thirteen-Lined	Mammals	Yes
Squirrels, Ground, Round-Tailed	Mammals	Yes
Mice, Deer, White-Footed	Mammals	Yes
Weasels (Other)	Mammals	Yes
Weasels, Long-Tailed	Mammals	Yes
Bats, Hoary	Mammals	Yes
Shrews (unadentified)	Mammals	Yes
Bats, Silver-Haired	Mammals	Yes
Woodrats, White-Throated	Mammals	Yes
Mice, Deer, North American	Mammals	Yes
Pronghorns (Antelope)	Mammals	Yes
Bats, Cave Myotis	Mammals	Yes
Squirrels, Ground, Belding`s	Mammals	Yes
Squirrels, Ground, Richardson`s	Mammals	Yes
Moose	Mammals	Yes
Bats, Pipistrelles, Western (Canyon Bat)	Mammals	Yes
Mice, Pocket, Arizona	Mammals	Yes
Shrews, Short-Tailed	Mammals	Yes
Rats, Kangaroo (unadentified)	Mammals	Yes
Bats, Lesser Long-Nosed	Mammals	Yes
Coatis	Mammals	Yes
Bats, Big Free Tail	Mammals	Yes

Moles, Eastern	Mammals	Yes
Prairie Dogs, White-Tailed	Mammals	Yes
Squirrels, Flying, Northern	Mammals	Yes
Hares, Jackrabbits, White-Tailed	Mammals	Yes
Weasels, Short Tailed	Mammals	Yes
Shrews, Least	Mammals	Yes
Shrews, Masked	Mammals	Yes
Deer, Red	Mammals	Yes
Rabbits, Cottontails, Mountain	Mammals	Yes
Squirrels, Flying, Southern	Mammals	Yes
Bats, California Myotis	Mammals	Yes
Woodrats, Bushy-Tailed	Mammals	Yes
Foxes, Swift	Mammals	Yes
Squirrels, Douglas	Mammals	Yes
Woodrats, Dusky Footed	Mammals	Yes
Squirrels, Ground, Mexican	Mammals	Yes
Bats, Pallid	Mammals	Yes
Skunks, Western Spotted	Mammals	Yes
Squirrels, Ground, Golden-Mantled	Mammals	Yes
Voles, Prairie	Mammals	Yes
Rats, Kangaroo, Ord`s	Mammals	Yes
Rabbits, Cottontails, New England	Mammals	Yes
Rats, Kangaroo, Merriam`s	Mammals	Yes
Squirrels, Ground, Columbian	Mammals	Yes
Squirrels, Ground, Wyoming	Mammals	Yes
Woodrats, Eastern	Mammals	Yes
Bats, Western Yellow	Mammals	Yes
Hares, Snowshoe	Mammals	Yes
Chipmunks (Other)	Mammals	Yes
Chipmunks, Cliff	Mammals	Yes
Bison (Buffalo)	Mammals	Yes
Deer, Sika	Mammals	Yes
Beavers, Mountain	Mammals	Yes
Mice, Deer, Cactus	Mammals	Yes
Squirrels, Ground, Uinta	Mammals	Yes
Bats, Bonnetted, Greatr (Westrn Mastiff)	Mammals	Yes
Bats, Western Red	Mammals	Yes
Woodrats, Southern Plains	Mammals	Yes
Bats, Fringed Myotis	Mammals	Yes
Foxes, Channel Island Gray	Mammals	Yes
Rats, Hutia	Mammals	Yes
Sheep, Bighorn	Mammals	Yes
Weasels, Least	Mammals	Yes
Bats, Yuma Myotis	Mammals	Yes
Lynx	Mammals	Yes
Sea Lions, Steller	Mammals	Yes
Wolves, Red	Mammals	Yes
Woodrats, Desert	Mammals	Yes

Bats, Arizona Myotis	Mammals	Yes
Bats, Long-Eared Myotis	Mammals	Yes
Foxes, Kit, San Joaquin (T/E)	Mammals	Yes
Martens, Pine	Mammals	Yes
Rabbits, Cottontails, Swamp	Mammals	Yes
Sea Lions, California	Mammals	Yes
Bats, Small-Footed Myotis	Mammals	Yes
Shrews, Pygmy	Mammals	Yes
Shrews, Short-Tailed, Northern	Mammals	Yes
Shrews, Short-Tailed, Southern	Mammals	Yes
Woodrats, Allegheny	Mammals	Yes
Bats, Big-Eared, Townsend`s	Mammals	Yes
Bats, Long-Tongued Mexican	Mammals	Yes
Bats, Pocketed Free-Tailed	Mammals	Yes
Caribou/Reindeer	Mammals	Yes
Muskrats, Round-Tailed	Mammals	Yes
Rabbits, Cottontails, Appalachian	Mammals	Yes
Rabbits, Cottontails, Brush Non-T/E	Mammals	Yes
Squirrels, Antelope, Harris`s	Mammals	Yes
Snakes, Brown Tree	Reptiles	No
Reptiles, Exotic	Reptiles	No
Iguanas, Green	Reptiles	No
Lizards, Monitor	Reptiles	No
Pythons, Burmese	Reptiles	No
Snakes, Non-Venomous (Other)	Reptiles	Yes
Snakes, Venomous (Other)	Reptiles	Yes
Snakes, Rattlesnakes, Western Diamond	Reptiles	Yes
Turtles, Common Snapping	Reptiles	Yes
Snakes, Gopher	Reptiles	Yes
Turtles (Other)	Reptiles	Yes
Snakes, Garter (All)	Reptiles	Yes
Lizards, (Other)	Reptiles	Yes
Snakes, Rattlesnakes, Black Tailed	Reptiles	Yes
Alligators, American	Reptiles	Yes
Snakes, Rattlesnakes, Mohave	Reptiles	Yes
Snakes, Rattlesnakes, Western	Reptiles	Yes
Turtles, Slider	Reptiles	Yes
Turtles, Painted	Reptiles	Yes
Salamanders (All)	Reptiles	Yes
Snakes, Bull	Reptiles	Yes
Snakes, Rattlesnakes, Prairie	Reptiles	Yes
Snakes, Rattlesnakes, Southern Pacific	Reptiles	Yes
Turtles, Common Map	Reptiles	Yes
Snakes, Coach Whip	Reptiles	Yes
Snakes, Common King	Reptiles	Yes
Snakes, Cottonmouth	Reptiles	Yes
Turtles, Red-Eared Slider	Reptiles	Yes
Snakes, Rattlesnakes, Red Diamond	Reptiles	Yes

Lizards, Gila Monster	Reptiles	Yes
Snakes, Rat, Western (Texas, Black)	Reptiles	Yes
Iguanas, Black Spiny-Tailed	Reptiles	Yes
Snakes, Sonoran Whipsnake	Reptiles	Yes
Turtles, Eastern Box	Reptiles	Yes
Turtles, Spiny Softshell	Reptiles	Yes
Snakes, Ring-Necked	Reptiles	Yes
Frogs/Toads (Other)	amphibians	Yes
Frogs, American Bullfrog	amphibians	Yes
